# Generation of multi-form exact wave solutions and linear stability analysis in the generalized (3+1)-D P-type plasma system using a modified extended mapping technique

**DOI:** 10.1038/s41598-026-49817-0

**Published:** 2026-05-14

**Authors:** Mohammed S. Ghayad, Hamdy M. Ahmed, Niveen M. Badra, Wafaa B. Rabie

**Affiliations:** 1 Department of Physics and Engineering Mathematics, Faculty of Engineering, Ain Shams University, Cairo, Egypt; 2 Department of Physics and Engineering Mathematics, Higher Institute of Engineering El Shorouk Academy, Cairo, Egypt; 3 Department of Mathematics, Faculty of Science Luxor University, Taiba, Luxor Egypt

**Keywords:** Structural waveform diversity, High-dimensional nonlinear system, Soliton solutions, Analytical methods, Elliptic wave structures, Linear stability analysis, Mathematics and computing, Optics and photonics, Physics

## Abstract

In this work, the wave solutions of the generalized (3+1)-dimensional P-type equation, a significant model for describing the evolution of waves in plasma physics, are investigated. The modified extended mapping method (MEMM) is applied as an effective analytical tool to get these solutions. Through the use of this method, a large variety of exact solutions is successfully derived, including Jacobi elliptic function solutions, bright and dark solitons, singular solitons, exponential forms, and singular periodic waveforms solutions. These solutions provide additional insight into the complex dynamics of the used equation. Furthermore, a linear stability analysis is performed to examine the stability of the steady-state solutions. The dispersion relation shows that the perturbation growth rate is purely imaginary for generic parameters, indicating neutral stability and the absence of modulation instability. Moreover, graphical representations of some of the solutions are given in order to disclose their physical behavior and better understand the corresponding wave phenomena.

## Introduction

Nonlinearity is a fundamental feature of nature, and many scientists believe that Nonlinear science is the major field in which to understand physical phenomena. The mathematical modeling of these phenomena is given in the form of Nonlinear partial differential equations (NLPDEs). Multi-dimensional NLPDEs have a vital role in representing intricate physical phenomena in many scientific fields such as quantum mechanics, fluid dynamics, optical fibers, and other fields. These equations frequently describe the development of quantities that vary with time and multiple spatial dimensions. Recent developments in wave propagation and Nonlinear optics have benefited from significant progress based on analytical and numerical methods, particularly in the study of soliton dynamics in various physical media. Clamond et al.^1^ establishes a comprehensive framework for generalized solitary waves with oscillatory tails and multi-pulse configurations, providing a useful perspective for interpreting the transition between periodic, elliptic, and localized solutions obtained in the present study. Algebraic construction techniques for singular solitary waves, as developed in^2^, further support the existence and characterization of solutions such as the $csc^2$ and $csch^2$ profiles reported here, while also informing their physical admissibility. Ghosh and Manda^3^ contrasted fiber Bragg grating optical filters for high-resolution sensing, outlining their application in engineering practice. Albayrak^4^ applied the methods of Kudryashov to recover optical solitons from Biswas–Milovic’s model of spatio-temporal dispersion, highlighting the efficiency of symbolic methods. Similarly, Qarni et al.^5^ applied the extended Adomian decomposition method to obtain closed-form solitons of the cubic-quartic Lakshmanan–Porsezian–Daniel equation, while Yıldırım^6^ dealt with the Manakov system soliton molecules employing the trial equation method.

On a different note, Kumar and Dhiman^7^ investigated compound forms like cone-shaped solitons and lumps using Lie symmetry and unification methods, while Li et al.^8^ demonstrated soliton resonance and bifurcation effects in the Maccari system. Wazwaz^9^ introduced new fourth-order integrable equations and constructed several soliton solutions, further distinguishing integrable systems. Niu et al^10^ employed the binary Darboux transformation for solving the A modified Kadomtsev-Petviashvili (MKP) equation. Comparative analyses of multi-peak and lump solutions by Kumar and Niwas^11^ once more demonstrated the stability of Lie group and unified methods towards variable-coefficient models. Moreover, related higher-order dispersive models with Hamiltonian structure^12^ and compact formulations supporting solitary wave interactions^13^ demonstrate that structurally similar nonlinear systems admit analogous families of localized, periodic, and singular waveforms. Also, the robustness and interaction properties of solitary waves in fully nonlinear dispersive systems have also been extensively studied^14^, offering further insight into the persistence and stability of the waveforms obtained in this work.

Other techniques, such as the modified extended direct algebraic method, were appropriately extended to other equations, such as the Nonlinear Schrödinger equation^15^, Salerno equation^16^, and fiber Bragg grating systems^17^. Subsequent works employed the advanced mapping and tanh-function methods of the analysis of complex Nonlinear equations like the Kundu–Eckhaus and RKL models, including various kinds of solitons and their modulated dynamics^18–21^. Collectively, these comprehensive studies highlight the power of present analysis techniques in the description of Nonlinear wave behavior in optical and physical systems.

In addition, efficient numerical methods for computing steady solitary waves^22^ and generalized solitary waves with oscillatory tails^23^ provide valuable tools for validating analytical solutions and exploring parameter–solution relationships in multidimensional settings. Further extensions and refined mathematical treatments of related nonlinear models have been presented in^24,25^.

Analyzing these equations and deriving their various solutions enables us to uncover the physical characteristics of the phenomenon. The (3 + 1)-dimensional B-type Kadomtsev–Petviashvili equation (BKPE) model is one of the multi-dimensional NLPDEs that has been studied through a considerable amount of research work^26–29^.

One of the significant models is a generalized (3 + 1)-dimensional P-type equation, which represents the evolution of plasma waves in plasma physics.

In the present research, we study the (3+1)-dimensional P-type equation model, which is given by the following equation^30^:

1 $$\begin{aligned} \mathcal {T} _{xxxy}+\mathfrak {a}_1 \mathcal {T} _{yt}+\mathfrak {a}_2 \left( \mathcal {T} \mathcal {T} _x\right) _y+\mathfrak {a}_3 \mathcal {T} _{xx}+\mathfrak {a}_4 \mathcal {T} _{zz}=0, \end{aligned}$$

where $\mathcal {T}=\mathcal {T}(x,y,z,t)$ models the propagation wave and ($\mathfrak {a}_1, \mathfrak {a}_2, \mathfrak {a}_3, \mathfrak {a}_4$) are real constants.

From a plasma physics perspective, the qualitative behavior of the nonlinear wave solutions is governed by the interplay between dispersion and nonlinear effects represented explicitly in Eq. (1). The term $\mathcal {T} _{xxxy}$ introduces higher-order mixed dispersion, which becomes significant in multidimensional wave propagation, while $\mathfrak {a}_1 \mathcal {T} _{yt}$ represents the temporal evolution coupled with transverse effects. The coefficients $a_1$ and $a_2$ correspond to second-order dispersion along the $x-$ and $z-$directions, respectively, and therefore control an isotropic wave spreading in the plasma medium. The nonlinear term $\mathfrak {a}_2 \left( \mathcal {T} \mathcal {T}_x\right) _y$ describes convective self-interaction and is responsible for wave steepening and energy localization.

The nature of the resulting wave structures depends critically on the relative signs and magnitudes of these coefficients. In particular, when the nonlinear coefficient $a_2$ and the dominant dispersion coefficient (primarily $a_3$ have the same sign $( a_2 /a_3)>0$, nonlinear steepening is balanced by dispersion, leading to the formation of localized compressive structures such as bright solitons and rogue-wave-type excitations. Conversely, when $(a_2 /a_3)<0$, the nonlinearity becomes effectively defocusing, and the system supports rarefactive (dark) solitons on a finite background. The coefficient $a_4$ introduces transverse dispersion along the $z-$ direction, which influences the three-dimensional stability and spatial spreading of the wave, while the mixed derivative term enhances directional coupling and can modify the propagation angle of localized structures.

In recent years, considerable attention has been devoted to the construction of exact travelling wave solutions for nonlinear evolution equations arising in plasma physics and related fields. In particular, high-dimensional models often exhibit a rich variety of coherent structures, including solitons, periodic waves, and singular solutions, which are essential for understanding complex wave dynamics. Although several analytical techniques, such as generalized exponential differential schemes, exp-expansion methods, and direct algebraic approaches, have been successfully applied to the generalized (3+1)-dimensional P-type equation, a systematic and unified framework for generating and organizing broad classes of solutions remains limited. Dhiman et al. succeeded in getting some exact solutions for (3+1)-dimensional P-type equation using the generalized exponential differential method^31^. While Senol et al.^32^ utilized exp(–$\phi (\xi )$)-expansion modified extended tanh-function methods to find exact solutions. Also Ghayad et al.^33^ examine the influence of the local M-fractional derivative on wave propagation in generalized (3+1)-dimensional P-type equation using the modified extended direct algebraic approach.

Motivated by the previous studies, the present work employs the modified extended mapping method (MEMM) as an effective and systematic tool to construct exact solutions of the considered model. To the best of our knowledge, this is the first application of MEMM to this class of equations. One of the main advantages of this approach lies in its ability to produce a wide spectrum of solution families within a unified formulation, while naturally revealing intrinsic relationships between key physical parameters such as amplitude, width, and wave speed. Singular periodic, (bright, dark, singular) soliton, exponential, and JE function solutions are the new exhibited solutions. Moreover, the obtained solutions are expressed in closed forms, allowing their limiting behavior to be consistently traced across different cases, thereby providing deeper insight into the underlying wave phenomena. Furthermore, contour, 2D, and 3D plots are presented for specific parameter values to demonstrate its realistic physical behavior. The structure of this work is classified as follows: Section Overview of the proposed methodology briefly describes the adopted methodology. Section Derivation of the model solutions provides the implementation of the proposed method. Section Linear stability analysis presents the linear stability analysis of the steady-state solutions. Section Graphical representation illustrates some solutions by graphical simulations. Finally, Section Conclusion concludes this study.

## Overview of the proposed methodology

This section introduces the fundamentals of the MEMM (see^34,35^). If we have the following NLPDE:

2 $$\begin{aligned} \textit{H} \left( \mathcal {T},~ \mathcal {T}_t,~ \mathcal {T}_{x},~\mathcal {T}_{y},~\mathcal {T}_{z},~ \mathcal {T}_{xx},~ \mathcal {T}_{yy},~\mathcal {T}_{zz},~\mathcal {T}_{xt},...\right) = 0. \end{aligned}$$

To solve the previous equation, we make the following assumption:

3 $$\begin{aligned} \mathcal {T}~(x,~y,~z,~t)=\mathcal {\textit{P}}(\varrho ), \qquad \qquad \qquad \varrho =\rho _1 x+\rho _2 y+\rho _3 z+\rho _4t. \end{aligned}$$

So, Eq. (2) can be redefined as next:

4 $$\begin{aligned} \textit{G} (\textit{P}, \textit{P}^\prime , \textit{P}^{\prime \prime }, \ldots ) = 0. \end{aligned}$$

Now, we can set the general solution to the previous equation as next,

5 $$\begin{aligned} \textit{P}(\varrho )=\sum _{i=0}^N \textit{f}_i \mathcal {L} ^i (\varrho )+\sum _{i=-1}^{-N} \textit{g}_{-i} \mathcal {L} ^i(\varrho )+\sum _{i=2}^N \textit{r}_i \mathcal {L} ^{i-2}(\varrho )\mathcal {L} '(\varrho )+\sum _{i=-1}^{-N} \textit{s}_{-i} \mathcal {L} ^i(\varrho )\mathcal {L} '(\varrho ), \end{aligned}$$

where $\mathcal {L}(\varrho )$ satisfy the next auxiliary equation:

6 $$\begin{aligned} \mathcal {L}'(\varrho )=\sqrt{\textbf{b}_0+\textbf{b}_1 \mathcal {L}(\varrho )+\textbf{b}_2 \mathcal {L}^2(\varrho )+\textbf{b}_3 \mathcal {L}^3(\varrho )+\textbf{b}_4 \mathcal {L}^4(\varrho )+\textbf{b}_6 \mathcal {L}^6(\varrho )}. \end{aligned}$$

The next cases are the solution of the previous equation:

**Case 1:** When $$\textbf{b}_0=~\textbf{b}_1=~\textbf{b}_3=\textbf{b}_6=0$$,

$$\begin{aligned} & \mathcal {L}(\varrho )=\sqrt{-\frac{\textbf{b}_2}{\textbf{b}_4}} \sec \left[ \varrho \sqrt{-\textbf{b}_2} \right] ,\quad \textbf{b}_2<0,~ \textbf{b}_4>0. \\ & \mathcal {L}(\varrho )=\sqrt{-\frac{\textbf{b}_2}{\textbf{b}_4}} \csc \left[ \varrho \sqrt{-\textbf{b}_2} \right] ,\quad \textbf{b}_2<0,~ \textbf{b}_4>0. \\ & \mathcal {L}(\varrho )=\sqrt{-\frac{\textbf{b}_2}{\textbf{b}_4}} \text {sech}\left[ \varrho \sqrt{\textbf{b}_2}\right] , \quad \textbf{b}_2>0,~ \textbf{b}_4<0. \end{aligned}$$

**Case 2:** When $$\textbf{b}_1=\textbf{b}_3=\textbf{b}_6=0,\textbf{b}_0=\frac{\textbf{b}_2^2}{4 \textbf{b}_4}$$:

$$\begin{aligned} & \mathcal {L}(\varrho )=\sqrt{\frac{-\textbf{b}_2}{2 \textbf{b}_4}} \tanh \left[ \varrho \sqrt{\frac{-\textbf{b}_2}{2}} \right] ,\quad \textbf{b}_2<0,~ \textbf{b}_4>0. \\ & \mathcal {L}(\varrho )=\sqrt{\frac{\textbf{b}_2}{2 \textbf{b}_4}} \tan \left[ \varrho \sqrt{\frac{\textbf{b}_2}{2}} \right] ,\quad ~~~~\textbf{b}_2>0, ~\textbf{b}_4>0. \end{aligned}$$

**Case 3:** When $$\textbf{b}_3=~\textbf{b}_4=~\textbf{b}_6=0$$:

$$\begin{aligned} \mathcal {L}(\varrho )=-\frac{\textbf{b}_1}{2 \textbf{b}_2}+e^{\varrho \sqrt{\textbf{b}_2} },~~~~~~~~\quad \textbf{b}_2>0,~ \textbf{b}_0=\frac{\textbf{b}_1^2}{4 \textbf{b}_2}. \end{aligned}$$

**Case 4:** When $$\textbf{b}_0=\textbf{b}_1=\textbf{b}_6=0$$,

$$\begin{aligned} & \mathcal {L}(\varrho )=- \frac{\textbf{b}_2}{\textbf{b}_3} \left( \tanh \left[ \frac{1}{2}\varrho \sqrt{\textbf{b}_2} \right] +1\right) ,~~~~~~\quad \textbf{b}_2>0,~\textbf{b}_4>0,~\textbf{b}_3=\pm 2 \sqrt{\textbf{b}_2 \textbf{b}_4}. \\ & \mathcal {L}(\varrho )=- \frac{\textbf{b}_2}{\textbf{b}_3} \left( \coth \left[ \frac{1}{2}\varrho \sqrt{\textbf{b}_2} \right] +1\right) ,~~~~~~~\quad \textbf{b}_2>0,~\textbf{b}_4>0,~\textbf{b}_3=\pm 2 \sqrt{\textbf{b}_2 \textbf{b}_4}. \\ & \mathcal {L}(\varrho )=\frac{\textbf{b}_2 {{\,\textrm{sech}\,}}^2\left[ \frac{1}{2} \varrho \sqrt{\textbf{b}_2} \right] }{2 \sqrt{\textbf{b}_2 \textbf{b}_4} \tanh \left[ \frac{1}{2}\varrho \sqrt{\textbf{b}_2} \right] -\textbf{b}_3},~~~~~\quad \textbf{b}_2>0,~ \textbf{b}_4>0,~\textbf{b}_3\ne \pm 2 \sqrt{\textbf{b}_2 \textbf{b}_4}. \\ & \mathcal {L}(\varrho )=\frac{\textbf{b}_2 \sec ^2\left[ \frac{1}{2} \varrho \sqrt{-\textbf{b}_2} \right] }{2 \sqrt{-\textbf{b}_2 \textbf{b}_4} \tan \left[ \frac{1}{2}\varrho \sqrt{-\textbf{b}_2} \right] +\textbf{b}_3},~~\quad \textbf{b}_2<0,~ \textbf{b}_4>0,~\textbf{b}_3\ne \pm 2 \sqrt{\textbf{b}_2 \textbf{b}_4}. \end{aligned}$$

**Case 5:** When $$\textbf{b}_1=\textbf{b}_3=0$$,

$$\begin{aligned} & \mathcal {L}(\varrho )=\sqrt{\frac{2 \textbf{b}_2 \text {sech}^2\left( \varrho \sqrt{\textbf{b}_2}\right) }{2 \sqrt{\textbf{b}_4^2-4 \textbf{b}_2 \textbf{b}_6}-\left( \sqrt{\textbf{b}_4^2-4 \textbf{b}_2 \textbf{b}_6}+\textbf{b}_4\right) \text {sech}^2\left( \varrho \sqrt{\textbf{b}_2}\right) }},~~~~~~~~~~~\quad \textbf{b}_2>0. \\ & \mathcal {L}(\varrho )=\sqrt{\frac{2 \textbf{b}_2 \sec ^2\left( \varrho \sqrt{-\textbf{b}_2}\right) }{2 \sqrt{\textbf{b}_4^2-4 \textbf{b}_2 \textbf{b}_6}-\left( \sqrt{\textbf{b}_4^2-4 \textbf{b}_2 \textbf{b}_6}-\textbf{b}_4\right) \sec ^2\left( \varrho \sqrt{-\textbf{b}_2}\right) }},~~~~~~~~~~~~~\textbf{b}_2<0. \end{aligned}$$

**Case 6:** When $$\textbf{b}_1=\textbf{b}_3=0 ~\text {and}~ 0\le \textit{m}\le 1$$,

**Table:** 

No.	$\textbf{b}_0$	$\textbf{b}_2$	$\textbf{b}_4$	$\mathcal {L}(\varrho )$
1	1	$-1-\textit{m}^2$	$\textit{m}^2$	$\text {sn}(\varrho ,\textit{m})$ or $cd(\varrho ,\textit{m})$
2	$\textit{m}^2-1$	$2-\textit{m}^2$	$-1$	$dn(\varrho ,\textit{m})$
3	$-\textit{m}^2$	$2\textit{m}^2-1$	$1-\textit{m}^2$	$nc(\varrho ,\textit{m})$
4	−1	$2-\textit{m}^2$	$\textit{m}^2-1$	$nd(\varrho ,\textit{m})$
5	$\textit{m}^4-2 \textit{m}^3+\textit{m}^2$	$-\frac{4}{\textit{m}}$	$-\textit{m}^2+6 \textit{m}-1$	$\frac{\textit{m} ~\text {cn}(\varrho |\textit{m}) \text {dn}(\varrho |\textit{m})}{c~ \text {sn}(\varrho |\textit{m})^2+1}$

To obtain the integer *N*, the balance principle is applied to the term with highest derivative and Nonlinear term of Eq. (4). Substituting Eqs.(5) and (6) into Eq. (4) which will give a polynomial in $\mathcal {L}(\varrho )$. Coefficients of identical-power terms are equated to zero. The algebraic equation system thus obtained is solved by Mathematica software.

## Derivation of the model solutions

This section introduces the implementation of MEMM on Eq.(1). We begin by applying the wave transformation introduced in Eq.(3) into Eq.(1), then it can be expressed as follows:

7 $$\begin{aligned} \rho _1^3 \rho _2 \textit{P}^{(4)}(\varrho )+\left( \mathfrak {a}_1 \rho _2 \rho _4+\mathfrak {a}_3 \rho _1^2+\mathfrak {a}_4 \rho _3^2\right) \textit{P}''(\varrho )+\mathfrak {a}_2 \rho _1 \rho _2 \left( \textit{P}'^2(\varrho )+\textit{P}(\varrho ) \textit{P}''(\varrho )\right) =0. \end{aligned}$$

Integrating Eq.(7) twice w.r.t. $\varrho$, where the integration constants are taken to be zero in accordance with the imposed boundary conditions. In particular, for localized solutions we assume

$$\begin{aligned} P(\varrho ) \rightarrow 0 \quad \text {and} \quad P^{(n)}(\varrho ) \rightarrow 0 \quad \text {as} \quad |\varrho | \rightarrow \infty , \end{aligned}$$

while for periodic solutions the constants can be set to zero without loss of generality by an appropriate normalization, then Eq.(7) will be as follow:

8 $$\begin{aligned} 2~\rho _1^3 \rho _2 \textit{P}''(\varrho )+2~\left( \mathfrak {a}_1 \rho _2 \rho _4+\mathfrak {a}_3 \rho _1^2+\mathfrak {a}_4 \rho _3^2\right) \textit{P}(\varrho )+\mathfrak {a}_2 \rho _1 \rho _2 \textit{P}^2(\varrho )=0. \end{aligned}$$

The balance principle is implied on Eq.(8) among $\textit{P}''(\varrho )$ and $\textit{P}^2(\varrho )$, hence the balance equation will be:

$$\begin{aligned} N+2=2N \end{aligned}$$

by solving this equation, we get $N=2$

Therefore, we can formulate the solution of Eq.(8) as next:

9 $$\begin{aligned} \textit{P}(\varrho )=\textit{f}_0+\textit{f}_1\; \mathcal {L}(\varrho )+\textit{f}_2 \;\mathcal {L}(\varrho )^2+\frac{\textit{g}_1}{\mathcal {L}(\varrho )}+\frac{\textit{g}_2}{\mathcal {L}(\varrho )^2}+\textit{r}_2 \;\mathcal {L} '(\varrho )+\mathfrak {s}_1\frac{ \mathcal {L} '(\varrho )}{\mathcal {L}(\varrho )}+\mathfrak {s}_2\frac{ \mathcal {L} '(\varrho )}{\mathcal {L}(\varrho )^2}, \end{aligned}$$

where $\textit{f} _0,\textit{f} _1~,\textit{f} _2,\textit{g}_1,\textit{g}_2,\textit{r}_2,\mathfrak {s} _1~\text {and}~\mathfrak {s}_2$ are constants that will be calculated, which $\textit{f} _2,\textit{g} _2,\textit{r}_2$ and $\mathfrak {s} _{2} \ne 0$ collectively.

Substituting Eq.(9) into Eq.(8) with the aid of Eq.(6), then setting the coefficients with common powers equal zero. to form Nonlinear system of equations Mathematica software. Hence, we obtained the solutions of Eq.(1)with the following cases. The nature of the obtained solutions (trigonometric versus hyperbolic) is governed by the sign of $\textbf{b}_2\textbf{b}_4$, while their physical admissibility depends additionally on the sign of the nonlinear coefficient $\mathfrak {a}_2$ under the condition $\mathfrak {a}_2\ne 0$.

***Case 1:*** $\textbf{b}_0=\textbf{b}_1=\textbf{b}_3=\textbf{b}_6=0$, we get $\mathfrak {s}_1= \mathfrak {s}_2= 0$ and

**(1.1)** $$\textit{f}_0= \textit{f}_1= 0,\textit{f}_2= -\frac{12~ \textbf{b}_4 ~\rho _1^2}{\mathfrak {a}_2},\textit{g}_1= \textit{g}_2= \textit{r}_2= 0,\rho _4=-\frac{\mathfrak {a}_3 \rho _1^2+\mathfrak {a}_4 \rho _3^2+4 b_2 \rho _2 \rho _1^3}{\mathfrak {a}_1 \rho _2}.$$

**(1.2)** $$\textit{f}_0=-\frac{8~ \textbf{b}_2~ \rho _1^2}{\mathfrak {a}_2},~ \textit{f}_1= 0,\textit{f}_2= -\frac{12~ \textbf{b}_4~ \rho _1^2}{\mathfrak {a}_2},\textit{g}_1= \textit{g}_2= \textit{r}_2= 0,\rho _4=-\frac{\mathfrak {a}_3 \rho _1^2+\mathfrak {a}_4 \rho _3^2-4 b_2 \rho _2 \rho _1^3}{\mathfrak {a}_1 \rho _2}.$$

Within the findings of result (1.1), solution of Eq.(1) shall be split into the following:

**(1.1.1)** When $\textbf{b}_2<0~\text {and}~\textbf{b}_4>0$ (i.e., $\textbf{b}_2\textbf{b}_4<0$), the resulting solutions exhibit a singular periodic form, as shown below:

10 $$\begin{aligned} \mathcal {T}_{1.1.1.a}=\frac{12~ \textbf{b}_2~ \rho _1^2}{\mathfrak {a}_2} ~\sec ^2 \left[ \;\left( \rho _1 x+\rho _2 y+\rho _3 z+\rho _4 t\right) \,\sqrt{-\textbf{b}_2}\,\right] , \end{aligned}$$

or

11 $$\begin{aligned} \mathcal {T}_{1.1.1.b}=\frac{12~ \textbf{b}_2~ \rho _1^2}{\mathfrak {a}_2} ~\csc ^2 \left[ \;\left( \rho _1 x+\rho _2 y+\rho _3 z+\rho _4 t\right) \,\sqrt{-\textbf{b}_2}\,\right] . \end{aligned}$$

These solutions are real-valued provided $\textbf{b}_2<0$, ensuring that $\sqrt{-\textbf{b}_2}\in \mathbb {R}$. The condition $\textbf{b}_2\textbf{b}_4<0$ leads to trigonometric-type solutions ($\sec ^2$, $\csc ^2$), which are periodic and singular. Due to their divergence at discrete points, these solutions are generally not physically admissible in standard plasma settings, but may describe limiting or strongly nonlinear regimes.

It is worth noting that some of the obtained solutions exhibit singular behavior. While these solutions are mathematically consistent within the applied method, they often correspond to limiting or non-physical regimes in plasma systems, such as wave breaking or unbounded growth. Therefore, their physical relevance is generally limited, and they should be interpreted primarily as analytical extensions of the solution space rather than directly observable waveforms.

**(1.1.2)** When $\textbf{b}_2>0~ \text {and}~\textbf{b}_4<0$ (i.e., $\textbf{b}_2\textbf{b}_4<0$), the resulting solution exhibits a bright soliton form, as shown below:

12 $$\begin{aligned} \mathcal {T}_{1.1.2}=\frac{12~ \textbf{b}_2~ \rho _1^2}{\mathfrak {a}_2} ~\text {sech}^2 \left[ \;\left( \rho _1 x+\rho _2 y+\rho _3 z+\rho _4 t\right) \,\sqrt{\textbf{b}_2}\,\right] . \end{aligned}$$

This solution is real-valued for $\textbf{b}_2>0$, ensuring $\sqrt{\textbf{b}_2}\in \mathbb {R}$. In this case, the sign of $\textbf{b}_2\textbf{b}_4<0$ yields hyperbolic-type solutions ($\text {sech}^2$), corresponding to localized structures. Moreover, for $\mathfrak {a}_2>0$, the amplitude is positive and the solution represents a physically admissible bright soliton, arising from the balance between nonlinearity and dispersion in the plasma system.

Within the findings of result (1.2), solution of Eq.(1) can be categorized as seen:

**(1.2.1)** When $\textbf{b}_2<0~\text {and}~\textbf{b}_4>0$ (i.e., $\textbf{b}_2\textbf{b}_4<0$), the resulting solution exhibits a singular periodic form, as shown below:

13 $$\begin{aligned} \mathcal {T}_{1.2.1.a}=-\frac{4~ \textbf{b}_2~ \rho _1^2}{\mathfrak {a}_2} \left( 2-3~\sec ^2 \left[ \;\left( \rho _1 x+\rho _2 y+\rho _3 z+\rho _4 t\right) \,\sqrt{-\textbf{b}_2}\,\right] \right) , \end{aligned}$$

or

14 $$\begin{aligned} \mathcal {T}_{1.2.1.b}=-\frac{4~ \textbf{b}_2~ \rho _1^2}{\mathfrak {a}_2} \left( 2-3~\csc ^2 \left[ \;\left( \rho _1 x+\rho _2 y+\rho _3 z+\rho _4 t\right) \,\sqrt{-\textbf{b}_2}\,\right] \right) . \end{aligned}$$

**(1.2.2)** When $\textbf{b}_2>0~ \text {and}~\textbf{b}_4<0$ (i.e., $\textbf{b}_2\textbf{b}_4<0$), the resulting solution exhibits a bright soliton form, as shown below:

15 $$\begin{aligned} \mathcal {T}_{1.2.2}=-\frac{4~ \textbf{b}_2~ \rho _1^2}{\mathfrak {a}_2} \left( 2-3~\text {sech}^2 \left[ \;\left( \rho _1 x+\rho _2 y+\rho _3 z+\rho _4 t\right) \,\sqrt{\textbf{b}_2}\,\right] \right) . \end{aligned}$$

***Case 2:*** $\textbf{b}_1=\textbf{b}_3=\textbf{b}_6=0$, we get $\mathfrak {s}_1= \mathfrak {s}_2= 0$ and

**(2.1)** $$\begin{aligned} & {f_0} = - \frac{{6\;{b_2}\;\rho _1^2}}{{{\mathfrak{a}_2}}},\;{f_1} = 0,{f_2} = - \frac{{12\;{b_4}\;\rho _1^2}}{{{\mathfrak{a}_2}}}, \\ & {g_1} = {g_2} = {r_2} = 0,{\rho _4} = - \frac{{{\mathfrak{a}_3}\rho _1^2 + {\mathfrak{a}_4}\rho _3^2 - 2{b_2}{\rho _2}\rho _1^3}}{{{\mathfrak{a}_1}{\rho _2}}}. \\ \end{aligned}$$

**(2.2)** $$\begin{aligned} & {f_0} = - \frac{{6\;{b_2}\;\rho _1^2}}{{{\mathfrak{a}_2}}},\;{f_1} = 0,{f_2} = 0,\; \\ & {g_1} = {r_2} = 0,\;{g_2} = - \frac{{3\;b_2^2\;\rho _1^2}}{{{\mathfrak{a}_2}\;{b_4}}},\; \\ & {\rho _4} = - \frac{{{\mathfrak{a}_3}\rho _1^2 + {\mathfrak{a}_4}\rho _3^2 - 2{b_2}{\rho _2}\rho _1^3}}{{{\mathfrak{a}_1}{\rho _2}}}. \\ \end{aligned}$$

**(2.3)** $$\begin{aligned} & {f_0} = - \frac{{12\;{b_2}\;\rho _1^2}}{{{\mathfrak{a}_2}}},\;{f_1} = 0,{f_2} = - \frac{{12\;{b_4}\;\rho _1^2}}{{{\mathfrak{a}_2}}},\;{g_1} = {r_2} = 0,\; \\ & {g_2} = - \frac{{3\;b_2^2\;\rho _1^2}}{{{\mathfrak{a}_2}\;{b_4}}},\;{\rho _4} = - \frac{{{\mathfrak{a}_3}\rho _1^2 + {\mathfrak{a}_4}\rho _3^2 - 8{b_2}{\rho _2}\rho _1^3}}{{{\mathfrak{a}_1}{\rho _2}}}. \\ \end{aligned}$$

Within the findings of result (2.1), solution of Eq.(1) can be categorized as seen:

**(2.1.1)** When $\textbf{b}_2<0~\text {and}~\textbf{b}_4>0$ (i.e., $\textbf{b}_2\textbf{b}_4<0$), the resulting solution exhibits a bright soliton form, as shown below:

16 $$\begin{aligned} \mathcal {T}_{2.1.1}=-\frac{6~ \textbf{b}_2~ \rho _1^2}{\mathfrak {a}_2} ~\text {sech}^2 \left[ \;\left( \rho _1 x+\rho _2 y+\rho _3 z+\rho _4 t\right) \,\sqrt{-\frac{\textbf{b}_2}{2}}\,\right] . \end{aligned}$$

**(2.1.2)** When $\textbf{b}_2>0~\text {and}~\textbf{b}_4>0$ (i.e., $\textbf{b}_2\textbf{b}_4>0$), the resulting solution exhibits a singular periodic form, as shown below:

17 $$\begin{aligned} \mathcal {T}_{2.1.2}=-\frac{6~ \textbf{b}_2~ \rho _1^2}{\mathfrak {a}_2} ~\sec ^2 \left[ \;\left( \rho _1 x+\rho _2 y+\rho _3 z+\rho _4 t\right) \,\sqrt{\frac{\textbf{b}_2}{2}}\,\right] . \end{aligned}$$

Within the findings of result (2.2), solution of Eq.(1) can be categorized as seen:

**(2.2.1)** When $\textbf{b}_2<0~\text {and}~\textbf{b}_4>0$ (i.e., $\textbf{b}_2\textbf{b}_4<0$), the resulting solution exhibits a singular soliton form, as shown below:

18 $$\begin{aligned} \mathcal {T}_{2.2.1}=\frac{6~ \textbf{b}_2~ \rho _1^2}{\mathfrak {a}_2} ~\text {csch}^2 \left[ \;\left( \rho _1 x+\rho _2 y+\rho _3 z+\rho _4 t\right) \,\sqrt{-\frac{\textbf{b}_2}{2}}\,\right] . \end{aligned}$$

**(2.2.2)** When $\textbf{b}_2>0~\text {and}~\textbf{b}_4>0$ (i.e., $\textbf{b}_2\textbf{b}_4>0$), the resulting solution exhibits a singular periodic form, as shown below:

19 $$\begin{aligned} \mathcal {T}_{2.2.2}=-\frac{6~ \textbf{b}_2~ \rho _1^2}{\mathfrak {a}_2} ~\csc ^2 \left[ \;\left( \rho _1 x+\rho _2 y+\rho _3 z+\rho _4 t\right) \,\sqrt{\frac{\textbf{b}_2}{2}}\,\right] . \end{aligned}$$

Within the findings of result (2.3), solution of Eq.(1) can be categorized as seen:

**(2.3.1)** When $\textbf{b}_2<0~\text {and}~\textbf{b}_4>0$ (i.e., $\textbf{b}_2\textbf{b}_4<0$), the resulting solution exhibits a singular soliton form, as shown below:

20 $$\begin{aligned} \mathcal {T}_{2.3.1}=\frac{24~ \textbf{b}_2~ \rho _1^2}{\mathfrak {a}_2} ~\text {csch}^2 \left[ 2 \;\left( \rho _1 x+\rho _2 y+\rho _3 z+\rho _4 t\right) \,\sqrt{-\frac{\textbf{b}_2}{2}}\,\right] . \end{aligned}$$

**(2.3.2)** If $\textbf{b}_2>0~\text {and}~\textbf{b}_4>0$ (i.e., $\textbf{b}_2\textbf{b}_4>0$), the resulting solution exhibits a singular periodic form, as shown below:

21 $$\begin{aligned} \mathcal {T}_{2.3.2}=-\frac{24~ \textbf{b}_2~ \rho _1^2}{\mathfrak {a}_2} ~\csc ^2 \left[ 2\;\left( \rho _1 x+\rho _2 y+\rho _3 z+\rho _4 t\right) \,\sqrt{\frac{\textbf{b}_2}{2}}\,\right] . \end{aligned}$$

***Case 3:*** If $\textbf{b}_3=\textbf{b}_4=\textbf{b}_6=0$, we get $\textit{f}_1=\textit{f}_2=\mathfrak {s}_1=\mathfrak {s}_2=0$ and

**(3.1)** $$\textit{f}_0=0,\textit{g}_1=-\frac{6~ \textbf{b}_1~ \rho _1^2}{\mathfrak {a}_2},~ \textit{g}_2=-\frac{12~ \textbf{b}_0~ \rho _1^2}{\mathfrak {a}_2},~ \textit{r}_2= 0,\rho _4=-\frac{-4 ~\mathfrak {a}_3~ \textbf{b}_0 ~\rho _1^2-4 ~\mathfrak {a}_4~ \textbf{b}_0 ~\rho _3^2+\textbf{b}_1^2 ~\rho _2 ~\rho _1^3}{4 ~\mathfrak {a}_1~\textbf{b}_0 ~\rho _2}.$$

**(3.2)** $$\textit{f}_0=-\frac{\textbf{b}_1^2 ~\rho _1^2}{2 ~\mathfrak {a}_2 ~\textbf{b}_0},~\textit{g}_1=-\frac{6~ \textbf{b}_1~ \rho _1^2}{\mathfrak {a}_2},~ \textit{g}_2=-\frac{12~ \textbf{b}_0~ \rho _1^2}{\mathfrak {a}_2},~ \textit{r}_2= 0,\rho _4=-\frac{-4 ~\mathfrak {a}_3~ \textbf{b}_0 ~\rho _1^2-4 ~\mathfrak {a}_4~ \textbf{b}_0 ~\rho _3^2-\textbf{b}_1^2 ~\rho _2 ~\rho _1^3}{4 ~\mathfrak {a}_1~\textbf{b}_0 ~\rho _2}.$$

Within the findings of result (3.1), solution of Eq.(1) can be categorized as seen:

**(3.1.1)** When $\textbf{b}_2>0~\text {and}~\textbf{b}_0=\frac{\textbf{b}_1^2}{4 \textbf{b}_2}$, the resulting solution exhibits an exponential form, as shown below:

22 $$\begin{aligned} \mathcal {T}_{3.1.1}=-\frac{24~\textbf{b}_1~ \textbf{b}_2^2~ \rho _1^2}{\mathfrak {a}_2}~\frac{e^{ \left( \rho _1 x+\rho _2 y+\rho _3 z+\rho _4 t\right) \sqrt{\textbf{b}_2}}}{\left( \textbf{b}_1-2 \textbf{b}_2 e^{\left( \rho _1 x+\rho _2 y+\rho _3 z+\rho _4 t\right) \sqrt{\textbf{b}_2}}\right) ^2}, \end{aligned}$$

where $$\left( \textbf{b}_1-2 \textbf{b}_2 e^{\left( \rho _1 x+\rho _2 y+\rho _3 z+\rho _4 t\right) \sqrt{\textbf{b}_2}}\right) \ne 0$$.

Within the findings of result (3.2), solution of Eq.(1) can be categorized as seen:

**(3.2.1)** When $\textbf{b}_2>0~\text {and}~\textbf{b}_0=\frac{\textbf{b}_1^2}{4 \textbf{b}_2}$, the resulting solution exhibits an exponential form, as shown below:

23 $$\begin{aligned} \mathcal {T}_{3.2.1}=-\frac{2~ \textbf{b}_2~ \rho _1^2}{\mathfrak {a}_2}~\left( 1+12~\textbf{b}_1~\textbf{b}_2~\frac{e^{ \left( \rho _1 x+\rho _2 y+\rho _3 z+\rho _4 t\right) \sqrt{\textbf{b}_2}}}{\left( \textbf{b}_1-2 \textbf{b}_2 e^{\left( \rho _1 x+\rho _2 y+\rho _3 z+\rho _4 t\right) \sqrt{\textbf{b}_2}}\right) ^2}\right) , \end{aligned}$$

where $$\left( \textbf{b}_1-2 \textbf{b}_2 e^{\left( \rho _1 x+\rho _2 y+\rho _3 z+\rho _4 t\right) \sqrt{\textbf{b}_2}}\right) \ne 0$$.

***Case 4:*** If $\textbf{b}_0=\textbf{b}_1=\textbf{b}_6=0$, we get $\mathfrak {s} _1=\mathfrak {s}_2=0,~\textit{f}_2= -\frac{12~ \textbf{b}_4~ \rho _1^2}{\mathfrak {a}_2}$ and

**(4.1)** $$\begin{aligned} & {f_0} = {f_1} = 0,\;{g_1} = {g_2} = {r_2} = {b_3} = 0,\; \\ & {\rho _4} = - \frac{{{\mathfrak{a}_3}\;\rho _1^2 + {\mathfrak{a}_4}\;\rho _3^2 + 4{b_2}\;{\rho _2}\;\rho _1^3}}{{{\mathfrak{a}_1}\;{\rho _2}}}. \\ \end{aligned}$$

**(4.2)** $$\begin{aligned} & {f_0} = - \frac{{8\;{b_2}\;\rho _1^2}}{{{\mathfrak{a}_2}}},\;{f_1} = 0,\;{g_1} = {g_2} = {r_2} = {b_3} = 0,\; \\ & {\rho _4} = - \frac{{{\mathfrak{a}_3}\;\rho _1^2 + {\mathfrak{a}_4}\;\rho _3^2 - 4{b_2}\;{\rho _2}\;\rho _1^3}}{{{\mathfrak{a}_1}\;{\rho _2}}}. \\ \end{aligned}$$

**(4.3)** $$\begin{aligned} & {f_0} = 0,\;{f_1} = \pm \frac{{12\rho _1^2\sqrt {{b_2}{b_4}} }}{{{\mathfrak{a}_2}}},\; \\ & {g_1} = {g_2} = {r_2} = 0,\;{b_3} = \mp 2\sqrt {{b_2}{b_4}} ,\; \\ & {\rho _4} = - \frac{{{\mathfrak{a}_3}\;\rho _1^2 + {\mathfrak{a}_4}\;\rho _3^2 + {b_2}\;{\rho _2}\;\rho _1^3}}{{{\mathfrak{a}_1}\;{\rho _2}}}. \\ \end{aligned}$$

**(4.4)** $$\begin{aligned} & {f_0} = - \frac{{2\;{b_2}\;\rho _1^2}}{{{\mathfrak{a}_2}}},\;{f_1} = \pm \frac{{12\rho _1^2\sqrt {{b_2}{b_4}} }}{{{\mathfrak{a}_2}}},\; \\ & {g_1} = {g_2} = {r_2} = 0,\;{b_3} = \mp 2\sqrt {{b_2}{b_4}} ,\; \\ & {\rho _4} = - \frac{{{\mathfrak{a}_3}\;\rho _1^2 + {\mathfrak{a}_4}\;\rho _3^2 - {b_2}\;{\rho _2}\;\rho _1^3}}{{{\mathfrak{a}_1}\;{\rho _2}}}. \\ \end{aligned}$$

Within the findings of result (4.1), solution of Eq.(1) can be categorized as seen:

**(4.1.1)** When $\textbf{b}_2>0,~\textbf{b}_4>0$ (i.e., $\textbf{b}_2\textbf{b}_4>0$) $\text {and}~\textbf{b}_3^2\ne 4 \textbf{b}_2 \textbf{b}_4$, the resulting solution exhibits a singular soliton form, as shown below:

24 $$\begin{aligned} \mathcal {T}_{4.1.1}=-\frac{12~ \textbf{b}_2~ \rho _1^2}{\mathfrak {a}_2} ~\text {csch}^2 \left[ \;\left( \rho _1 x+\rho _2 y+\rho _3 z+\rho _4 t\right) \,\sqrt{\textbf{b}_2}\,\right] . \end{aligned}$$

**(4.1.2)** When $\textbf{b}_2<0,~\textbf{b}_4>0$ (i.e., $\textbf{b}_2\textbf{b}_4<0$)$~\text {and}~\textbf{b}_3^2\ne 4 \textbf{b}_2 \textbf{b}_4$, the resulting solution exhibits a singular periodic form, as shown below:

25 $$\begin{aligned} \mathcal {T}_{4.1.2}=\frac{12~ \textbf{b}_2~ \rho _1^2}{\mathfrak {a}_2} ~\csc ^2 \left[ \;\left( \rho _1 x+\rho _2 y+\rho _3 z+\rho _4 t\right) \,\sqrt{-\textbf{b}_2}\,\right] . \end{aligned}$$

Within the findings of result (4.2), solution of Eq.(1) can be categorized as seen:

**(4.2.1)** When $\textbf{b}_2>0,~\textbf{b}_4>0$ (i.e., $\textbf{b}_2\textbf{b}_4>0$)$~\text {and}~\textbf{b}_3^2\ne 4 \textbf{b}_2 \textbf{b}_4$, the resulting solution exhibits a singular soliton form, as shown below:

26 $$\begin{aligned} \mathcal {T}_{4.2.1}=-\frac{4~ \textbf{b}_2~ \rho _1^2}{\mathfrak {a}_2}\left( 2+3 ~\text {csch}^2 \left[ \;\left( \rho _1 x+\rho _2 y+\rho _3 z+\rho _4 t\right) \,\sqrt{\textbf{b}_2}\,\right] \right) . \end{aligned}$$

**(4.2.2)** When $\textbf{b}_2<0,~\textbf{b}_4>0$ (i.e., $\textbf{b}_2\textbf{b}_4<0$) $\text {and}~\textbf{b}_3^2\ne 4 \textbf{b}_2 \textbf{b}_4$, the resulting solution exhibits a singular periodic form, as shown below:

27 $$\begin{aligned} \mathcal {T}_{4.2.2}=-\frac{4~ \textbf{b}_2~ \rho _1^2}{\mathfrak {a}_2} ~\left( 2-3\csc ^2 \left[ \;\left( \rho _1 x+\rho _2 y+\rho _3 z+\rho _4 t\right) \,\sqrt{-\textbf{b}_2}\,\right] \right) . \end{aligned}$$

Within the findings of result (4.3), solution of Eq.(1) can be categorized as seen:

**(4.3.1)** When $\textbf{b}_2>0,~\textbf{b}_4>0$ (i.e., $\textbf{b}_2\textbf{b}_4>0$) $\text {and}~\textbf{b}_3^2=4 \textbf{b}_2 \textbf{b}_4$, the resulting solutions exhibit a bright and dark soliton form, as shown below:

28 $$\begin{aligned} \mathcal {T}_{4.3.1.a}=\frac{3~ \textbf{b}_2~ \rho _1^2}{\mathfrak {a}_2} ~\text {sech}^2 \left[ \;\frac{1}{2}\left( \rho _1 x+\rho _2 y+\rho _3 z+\rho _4 t\right) \,\sqrt{\textbf{b}_2}\,\right] , \end{aligned}$$

or

29 $$\begin{aligned} \mathcal {T}_{4.3.1.b}=\frac{3~ \textbf{b}_2~ \rho _1^2}{\mathfrak {a}_2}\left( 3+4 \text {tanh} \left[ \;\frac{1}{2}\varrho \,\sqrt{\textbf{b}_2}\,\right] +\tanh ^2 \left[ \;\frac{1}{2}\varrho \,\sqrt{\textbf{b}_2}\,\right] \right) , \end{aligned}$$

where $$\varrho =\left( \rho _1 x+\rho _2 y+\rho _3 z+\rho _4 t\right)$$.

**(4.3.2)** When $\textbf{b}_2>0,~\textbf{b}_4>0$ (i.e., $\textbf{b}_2\textbf{b}_4>0$) $\text {and}~\textbf{b}_3^2=4 \textbf{b}_2 \textbf{b}_4$, the resulting solutions exhibit the singular soliton form, as shown below:

30 $$\begin{aligned} \mathcal {T}_{4.3.2.a}=-\frac{3~ \textbf{b}_2~ \rho _1^2}{\mathfrak {a}_2} ~\text {csch}^2 \left[ \;\frac{1}{2}\left( \rho _1 x+\rho _2 y+\rho _3 z+\rho _4 t\right) \,\sqrt{\textbf{b}_2}\,\right] , \end{aligned}$$

or

31 $$\begin{aligned} \mathcal {T}_{4.3.2.b}=\frac{3~ \textbf{b}_2~ \rho _1^2}{\mathfrak {a}_2}\left( 3+4 \text {coth} \left[ \;\frac{1}{2}\varrho \,\sqrt{\textbf{b}_2}\,\right] +\coth ^2 \left[ \;\frac{1}{2}\varrho \,\sqrt{\textbf{b}_2}\,\right] \right) , \end{aligned}$$

where $$\varrho =\left( \rho _1 x+\rho _2 y+\rho _3 z+\rho _4 t\right)$$.

Within the findings of result (4.4), solution of Eq.(1) can be categorized as seen:

**(4.4.1)** When $\textbf{b}_2>0,~\textbf{b}_4>0$ (i.e., $\textbf{b}_2\textbf{b}_4>0$) $\text {and}~\textbf{b}_3^2=4 \textbf{b}_2 \textbf{b}_4$, the resulting solutions exhibit a bright and dark soliton form, as shown below:

32 $$\begin{aligned} \mathcal {T}_{4.4.1.a}=-\frac{\textbf{b}_2~ \rho _1^2}{\mathfrak {a}_2} ~\left( 2-3\text {sech}^2 \left[ \;\frac{1}{2}\left( \rho _1 x+\rho _2 y+\rho _3 z+\rho _4 t\right) \,\sqrt{\textbf{b}_2}\,\right] \right) , \end{aligned}$$

or

33 $$\begin{aligned} \mathcal {T}_{4.4.1.b}=\frac{\textbf{b}_2~ \rho _1^2}{\mathfrak {a}_2}\left( 7+12 \text {tanh} \left[ \;\frac{1}{2}\varrho \,\sqrt{\textbf{b}_2}\,\right] +\tanh ^2 \left[ \;\frac{1}{2}\varrho \,\sqrt{\textbf{b}_2}\,\right] \right) , \end{aligned}$$

where $$\varrho =\left( \rho _1 x+\rho _2 y+\rho _3 z+\rho _4 t\right)$$.

**(4.4.2)** When $\textbf{b}_2>0,~\textbf{b}_4>0$ (i.e., $\textbf{b}_2\textbf{b}_4>0$) $\text {and}~\textbf{b}_3^2=4 \textbf{b}_2 \textbf{b}_4$, the resulting solutions exhibit the singular soliton form, as shown below:

34 $$\begin{aligned} \mathcal {T}_{4.4.2.a}=-\frac{ \textbf{b}_2~ \rho _1^2}{\mathfrak {a}_2} ~\left( 2+3\text {csch}^2 \left[ \;\frac{1}{2}\left( \rho _1 x+\rho _2 y+\rho _3 z+\rho _4 t\right) \,\sqrt{\textbf{b}_2}\,\right] \right) , \end{aligned}$$

or

35 $$\begin{aligned} \mathcal {T}_{4.4.2.b}=\frac{ \textbf{b}_2~ \rho _1^2}{\mathfrak {a}_2}\left( 7+12 \text {coth} \left[ \;\frac{1}{2}\varrho \,\sqrt{\textbf{b}_2}\,\right] +\coth ^2 \left[ \;\frac{1}{2}\varrho \,\sqrt{\textbf{b}_2}\,\right] \right) , \end{aligned}$$

where $$\varrho =\left( \rho _1 x+\rho _2 y+\rho _3 z+\rho _4 t\right)$$.

***Case 5:*** If $\textbf{b}_1=\textbf{b}_3=0$, we get $\mathfrak {s}_1=\mathfrak {s}_2= \textit{r}_2=0$ and

$$\textit{f}_0=-\frac{4 \rho _1^2 \left( \textbf{b}_2\pm \sqrt{\textbf{b}_2^2-3 \textbf{b}_0 \textbf{b}_4}\right) }{a_2},~\textit{f}_1=\textit{f}_2= \textit{g}_1=0,~ \textit{g}_2=-\frac{12~ \textbf{b}_0~ \rho _1^2}{\mathfrak {a}_2},\rho _4=-\frac{\mathfrak {a}_3 \rho _1^2\mp 4 \rho _1^3 \rho _2\sqrt{\textbf{b}_2^2-3 \textbf{b}_0 \textbf{b}_4} +\mathfrak {a}_4 \rho _3^2}{\mathfrak {a}_1 \rho _2}.$$

Under the outcomes this case, solution of Eq.(1) can be categorized as seen:

**(5.1)** When $\textbf{b}_2>0$,the resulting solution exhibits a hyperbolic form, as shown below:

36 $$\begin{aligned} \mathcal {T}_{5.1}=-\frac{ 4~ \rho _1^2}{\mathfrak {a}_2}\left( \textbf{b}_2+ \sqrt{\textbf{b}_2^2-3 \textbf{b}_0 \textbf{b}_4}+\frac{3 \textbf{b}_0}{2 \textbf{b}_2}\left( -b_4+\sqrt{\textbf{b}_4^2-4 \textbf{b}_2 \textbf{b}_6} ~\text {cosh}\left[ 2 \varrho \sqrt{\textbf{b}_2} \right] \right) \right) , \end{aligned}$$

where $$\varrho =\left( \rho _1 x+\rho _2 y+\rho _3 z+\rho _4 t\right)$$.

**(5.2)** When $\textbf{b}_2<0$,the resulting solution exhibits a periodic form, as shown below:

37 $$\begin{aligned} \mathcal {T}_{5.2}=-\frac{ 4~ \rho _1^2}{\mathfrak {a}_2}\left( \textbf{b}_2+\sqrt{\textbf{b}_2^2-3 \textbf{b}_0 \textbf{b}_4}+\frac{3 \textbf{b}_0}{2 \textbf{b}_2}\left( -b_4+\sqrt{\textbf{b}_4^2-4 \textbf{b}_2 \textbf{b}_6}~ \cos \left[ 2 \varrho \sqrt{-\textbf{b}_2} \right] \right) \right) , \end{aligned}$$

where $$\varrho =\left( \rho _1 x+\rho _2 y+\rho _3 z+\rho _4 t\right)$$.

***Case 6:*** If $\textbf{b}_1=\textbf{b}_3=\textbf{b}_6=0$, we get $\textit{f}_1=\textit{g}_1=\mathfrak {s}_1=\mathfrak {s}_2= \textit{r}_2=0$ and

**(6.1)** $$\begin{aligned} & {f_0} = - \frac{{4\rho _1^2\left( {{b_2} - \sqrt {b_2^2 - 3{b_0}{b_4}} } \right)}}{{{a_2}}},\;{f_2} = 0,\;{g_2} = - \frac{{12\;{b_0}\;\rho _1^2}}{{{\mathfrak{a}_2}}}, \\ & {\rho _4} = - \frac{{{\mathfrak{a}_3}\rho _1^2 \mp 4\rho _1^3{\rho _2}\sqrt {b_2^2 - 3{b_0}{b_4}} + {\mathfrak{a}_4}\rho _3^2}}{{{\mathfrak{a}_1}{\rho _2}}}. \\ \end{aligned}$$

**(6.2)** $$\begin{aligned} & {f_0} = - \frac{{4\rho _1^2\left( {{b_2} - \sqrt {b_2^2 - 3{b_0}{b_4}} } \right)}}{{{a_2}}},\;{g_2} = 0,\;{f_2} = - \frac{{12\;{b_4}\;\rho _1^2}}{{{\mathfrak{a}_2}}},\; \\ & {\rho _4} = - \frac{{{\mathfrak{a}_3}\rho _1^2 \mp 4\rho _1^3{\rho _2}\sqrt {b_2^2 - 3{b_0}{b_4}} + {\mathfrak{a}_4}\rho _3^2}}{{{\mathfrak{a}_1}{\rho _2}}}. \\ \end{aligned}$$

**(6.3)** $$\begin{aligned} & {f_0} = - \frac{{4\rho _1^2\left( {{b_2} - \sqrt {b_2^2 + 12{b_0}{b_4}} } \right)}}{{{a_2}}},\; \\ & {f_2} = - \frac{{12\;{b_4}\;\rho _1^2}}{{{\mathfrak{a}_2}}},\;{g_2} = - \frac{{12\;{b_0}\;\rho _1^2}}{{{\mathfrak{a}_2}}}, \\ & {\rho _4} = - \frac{{{\mathfrak{a}_3}\rho _1^2 \mp 4\rho _1^3{\rho _2}\sqrt {b_2^2 + 12{b_0}{b_4}} + {\mathfrak{a}_4}\rho _3^2}}{{{\mathfrak{a}_1}{\rho _2}}}. \\ \end{aligned}$$

Within the findings of result (6.1), solution of Eq.(1) can be categorized as seen:

**(6.1.1)** If $\textbf{b}_0=1,\textbf{b}_2=-\textit{m}^2-1~\text {and}~\textbf{b}_4=\textit{m}^2$, the resulting solutions exhibit a Jacobi elliptic(JE) function form, as shown below:

38 $$\begin{aligned} \mathcal {T}_{6.1.1.a}=-\frac{ 4~ \rho _1^2}{\mathfrak {a}_2}\left( -1-\textit{m}^2- \sqrt{\textit{m}^4-\textit{m}^2+1}+3~ \text {ns}^2[\rho _1 x+\rho _2 y+\rho _3 z+\rho _4 t ]\right) , \end{aligned}$$

or

39 $$\begin{aligned} \mathcal {T}_{6.1.1.b}=-\frac{ 4~ \rho _1^2}{\mathfrak {a}_2}\left( -1-\textit{m}^2- \sqrt{\textit{m}^4-\textit{m}^2+1}+3~ \text {dc}^2[\rho _1 x+\rho _2 y+\rho _3 z+\rho _4 t ]\right) , \end{aligned}$$

where $$0\le \textit{m}\le 1$$.

When setting $\textit{m}=1$ in Eq.(38), the resulting solution exhibits a singular soliton nature, as shown below:

40 $$\begin{aligned} \mathcal {T}_{6.1.1.1}=-\frac{12~ \rho _1^2}{\mathfrak {a}_2} ~\text {csch}^2 \left[ \;\rho _1 x+\rho _2 y+\rho _3 z+\rho _4 t\,\right] . \end{aligned}$$

When setting $\textit{m}=0$ in Eq.(38) and Eq.(39) respectively, the resulting solutions exhibit a singular periodic nature, as shown below:

41 $$\begin{aligned} \mathcal {T}_{6.1.1.2}=-\frac{12~ \rho _1^2}{\mathfrak {a}_2} ~\csc ^2 \left[ \;\rho _1 x+\rho _2 y+\rho _3 z+\rho _4 t\,\right] , \end{aligned}$$

and

42 $$\begin{aligned} \mathcal {T}_{6.1.1.2}=-\frac{12~ \rho _1^2}{\mathfrak {a}_2} ~\sec ^2 \left[ \;\rho _1 x+\rho _2 y+\rho _3 z+\rho _4 t\,\right] . \end{aligned}$$

**(6.1.2)** If $\textbf{b}_0=\textit{m}^2-1,\textbf{b}_2=2-\textit{m}^2~\text {and}~\textbf{b}_4=-1$, the resulting solution exhibits a JE function nature, as shown below:

43 $$\begin{aligned} \mathcal {T}_{6.1.2}=-\frac{ 4~ \rho _1^2}{\mathfrak {a}_2}\left( 2-\textit{m}^2- \sqrt{\textit{m}^4-\textit{m}^2+1}+3~ (\textit{m}^2-1)\;\text {nd}^2[\rho _1 x+\rho _2 y+\rho _3 z+\rho _4 t ]\right) , \end{aligned}$$

where $$0\le \textit{m}< 1$$.

**(6.1.3)** If $\textbf{b}_0=-\textit{m}^2,\textbf{b}_2=-1+2\textit{m}^2~\text {and}~\textbf{b}_4=1-\textit{m}^2$, the resulting solution exhibits a JE function form, as shown below:

44 $$\begin{aligned} \mathcal {T}_{6.1.3}=-\frac{ 4~ \rho _1^2}{\mathfrak {a}_2}\left( -1+2\textit{m}^2- \sqrt{\textit{m}^4-\textit{m}^2+1}-3~\textit{m}^2\text {cn}^2[\rho _1 x+\rho _2 y+\rho _3 z+\rho _4 t ]\right) , \end{aligned}$$

where $$0< \textit{m}\le 1$$.

When setting $\textit{m}=1$ in Eq.(44), the resulting solution exhibits a bright soliton form, as shown below:

45 $$\begin{aligned} \mathcal {T}_{6.1.3.1}=\frac{12~ \rho _1^2}{\mathfrak {a}_2} ~\text {sech}^2 \left[ \;\rho _1 x+\rho _2 y+\rho _3 z+\rho _4 t\,\right] . \end{aligned}$$

**(6.1.4)** If $\textbf{b}_0=-1,\textbf{b}_2=2-\textit{m}^2~\text {and}~\textbf{b}_4=\textit{m}^2-1$, the resulting solution exhibits a JE function form, as shown below:

46 $$\begin{aligned} \mathcal {T}_{6.1.4}=-\frac{ 4~ \rho _1^2}{\mathfrak {a}_2}\left( 2-\textit{m}^2- \sqrt{\textit{m}^4-\textit{m}^2+1}-3~\text {dn}^2[\rho _1 x+\rho _2 y+\rho _3 z+\rho _4 t ]\right) , \end{aligned}$$

where $$0\le \textit{m}\le 1$$.

**(6.1.5)** If $\textbf{b}_0=\frac{1}{4},\textbf{b}_2=\frac{1}{2}(\textit{m}^2-2)~\text {and}~\textbf{b}_4=\frac{\textit{m}^4}{4}$, the resulting solution exhibits a JE function form, as shown below:

47 $$\begin{aligned} \mathcal {T}_{6.1.5}=-\frac{ \rho _1^2}{\mathfrak {a}_2}\left( -4+2\textit{m}^2\pm \sqrt{\textit{m}^4-16\textit{m}^2+16}+\frac{3~ (1+\text {dn}[\rho _1 x+\rho _2 y+\rho _3 z+\rho _4 t ])^2}{\text {sn}^2[\rho _1 x+\rho _2 y+\rho _3 z+\rho _4 t]}\right) , \end{aligned}$$

where $$0\le \textit{m}\le 1$$.

When setting $\textit{m}=1$ in Eq.(47), the resulting solution exhibits a singular soliton form, as shown below:

48 $$\begin{aligned} \mathcal {T}_{6.1.5.1}=-\frac{3~ \rho _1^2}{\mathfrak {a}_2} ~\text {csch}^2 \left[ \;\frac{1}{2}(\rho _1 x+\rho _2 y+\rho _3 z+\rho _4 t\,)\right] . \end{aligned}$$

Within the findings of result (6.2), solution of Eq.(1) can be categorized as seen:

**(6.2.1)** If $\textbf{b}_0=1,\textbf{b}_2=-\textit{m}^2-1~\text {and}~\textbf{b}_4=\textit{m}^2$, the resulting solutions exhibit a JE function form, as shown below:

49 $$\begin{aligned} \mathcal {T}_{6.2.1}=-\frac{ 4~ \rho _1^2}{\mathfrak {a}_2}\left( -1-\textit{m}^2- \sqrt{\textit{m}^4-\textit{m}^2+1}+3\textit{m}^2~ \text {sn}^2[\rho _1 x+\rho _2 y+\rho _3 z+\rho _4 t ]\right) , \end{aligned}$$

or

50 $$\begin{aligned} \mathcal {T}_{6.2.1}=-\frac{ 4~ \rho _1^2}{\mathfrak {a}_2}\left( -1-\textit{m}^2- \sqrt{\textit{m}^4-\textit{m}^2+1}+3\textit{m}^2~ \text {cd}^2[\rho _1 x+\rho _2 y+\rho _3 z+\rho _4 t ]\right) , \end{aligned}$$

where $$0< \textit{m}\le 1$$.

**(6.2.2)** If $\textbf{b}_0=\textit{m}^2-1,\textbf{b}_2=2-\textit{m}^2~\text {and}~\textbf{b}_4=-1$, the resulting solution exhibits a JE function form, as shown below:

51 $$\begin{aligned} \mathcal {T}_{6.2.2}=-\frac{ 4~ \rho _1^2}{\mathfrak {a}_2}\left( 2-\textit{m}^2- \sqrt{\textit{m}^4-\textit{m}^2+1}-3~ \text {dn}^2[\rho _1 x+\rho _2 y+\rho _3 z+\rho _4 t ]\right) , \end{aligned}$$

where $$0\le \textit{m}\le 1$$.

**(6.2.3)** If $\textbf{b}_0=-\textit{m}^2,\textbf{b}_2=-1+2\textit{m}^2~\text {and}~\textbf{b}_4=1-\textit{m}^2$,the resulting solution exhibits a JE function form, as shown below:

52 $$\begin{aligned} \mathcal {T}_{6.2.3}=-\frac{ 4~ \rho _1^2}{\mathfrak {a}_2}\left( -1+2\textit{m}^2- \sqrt{\textit{m}^4-\textit{m}^2+1}+3(1-\textit{m}^2)~\text {nc}^2[\rho _1 x+\rho _2 y+\rho _3 z+\rho _4 t ]\right) , \end{aligned}$$

where $$0\le \textit{m}< 1$$.

**(6.2.4)** If $\textbf{b}_0=-1,\textbf{b}_2=2-\textit{m}^2~\text {and}~\textbf{b}_4=\textit{m}^2-1$, the resulting solution exhibits a JE function form, as shown below:

53 $$\begin{aligned} \mathcal {T}_{6.2.4}=-\frac{ 4~ \rho _1^2}{\mathfrak {a}_2}\left( 2-\textit{m}^2-\sqrt{\textit{m}^4-\textit{m}^2+1}+3(-1+\textit{m}^2)~\text {nd}^2[\rho _1 x+\rho _2 y+\rho _3 z+\rho _4 t ]\right) , \end{aligned}$$

where $$0\le \textit{m}< 1$$.

**(6.2.5)** If $\textbf{b}_0=\frac{1}{4},\textbf{b}_2=\frac{1}{2}(\textit{m}^2-2)~\text {and}~\textbf{b}_4=\frac{\textit{m}^4}{4}$, the resulting solution exhibits a JE function form, as shown below:

54 $$\begin{aligned} \mathcal {T}_{6.2.5}=-\frac{ \rho _1^2}{\mathfrak {a}_2}\left( -4+2\textit{m}^2- \sqrt{\textit{m}^4-16\textit{m}^2+16}+\frac{3\textit{m}^4~\text {sn}^2[\rho _1 x+\rho _2 y+\rho _3 z+\rho _4 t]}{ (1+\text {dn}[\rho _1 x+\rho _2 y+\rho _3 z+\rho _4 t ])^2}\right) , \end{aligned}$$

where $$0< \textit{m}\le 1$$.

When setting $\textit{m}=1$ in Eq.(54), the resulting solution exhibits a bright soliton form, as shown below:

55 $$\begin{aligned} \mathcal {T}_{6.2.5.1}=\frac{3~ \rho _1^2}{\mathfrak {a}_2} \text {sech}^2 \left[ \;\frac{1}{2}\left( \rho _1 x+\rho _2 y+\rho _3 z+\rho _4 t\right) \,\right] . \end{aligned}$$

Within the findings of result (6.3), solution of Eq.(1) can be categorized as seen:

**(6.3.1)** If $\textbf{b}_0=1,\textbf{b}_2=-\textit{m}^2-1~\text {and}~\textbf{b}_4=\textit{m}^2$, the resulting solutions exhibit a JE function form, as shown below:

56 $$\begin{aligned} \mathcal {T}_{6.3.1}=-\frac{ 4~ \rho _1^2}{\mathfrak {a}_2}\left( -1-\textit{m}^2- \sqrt{\textit{m}^4-\textit{m}^2+1}+3\textit{m}^2~ \text {sn}^2[\varrho ]+3~\text {ns}^2[\varrho ]\right) , \end{aligned}$$

or

57 $$\begin{aligned} \mathcal {T}_{6.3.1}=-\frac{ 4~ \rho _1^2}{\mathfrak {a}_2}\left( -1-\textit{m}^2- \sqrt{\textit{m}^4-\textit{m}^2+1}+3\textit{m}^2~ \text {cd}^2[\varrho ]+3~\text {dc}^2[\varrho ]\right) , \end{aligned}$$

where $$\varrho =\left( \rho _1 x+\rho _2 y+\rho _3 z+\rho _4 t\right)$$ and $$0\le \textit{m}\le 1$$.

When setting $\textit{m}=1$ in Eq.(57), a the resulting solution exhibits a singular soliton form, as shown below:

58 $$\begin{aligned} \mathcal {T}_{6.3.1.1}=-\frac{4~ \rho _1^2}{\mathfrak {a}_2}\left( 5+12 ~\text {csch}^2 \left[ 2(\;\rho _1 x+\rho _2 y+\rho _3 z+\rho _4 t)\,\right] \,\right) . \end{aligned}$$

**(6.3.2)** If $\textbf{b}_0=\textit{m}^2-1,\textbf{b}_2=2-\textit{m}^2~\text {and}~\textbf{b}_4=-1$, the resulting solution exhibits a JE function form, as shown below:

59 $$\begin{aligned} \mathcal {T}_{6.3.2}=-\frac{ 4~ \rho _1^2}{\mathfrak {a}_2}\left( 2-\textit{m}^2- \sqrt{\textit{m}^4-\textit{m}^2+1}+3~(-1+\textit{m}^2)~ \text {nd}^2[\varrho ]-3~\text {dn}^2[\varrho ]\right) , \end{aligned}$$

where $$\varrho =\left( \rho _1 x+\rho _2 y+\rho _3 z+\rho _4 t\right)$$ and $$0\le \textit{m}\le 1$$.

**(6.3.3)** If $\textbf{b}_0=-\textit{m}^2,\textbf{b}_2=-1+2\textit{m}^2~\text {and}~\textbf{b}_4=1-\textit{m}^2$, the resulting solution exhibits a JE function form, as shown below:

60 $$\begin{aligned} \mathcal {T}_{6.3.3}=-\frac{ 4~ \rho _1^2}{\mathfrak {a}_2}\left( -1+2\textit{m}^2- \sqrt{\textit{m}^4-\textit{m}^2+1}-3~\textit{m}^2~ \text {cn}^2[\varrho ]+3~(1-\textit{m}^2)~\text {nc}^2[\varrho ]\right) , \end{aligned}$$

where $$\varrho =\left( \rho _1 x+\rho _2 y+\rho _3 z+\rho _4 t\right)$$ and $$0\le \textit{m}\le 1$$.

**(6.3.4)** If $\textbf{b}_0=-1,\textbf{b}_2=2-\textit{m}^2~\text {and}~\textbf{b}_4=\textit{m}^2-1$, the resulting solution exhibits a JE function form, as shown below:

61 $$\begin{aligned} \mathcal {T}_{6.3.4}=-\frac{ 4~ \rho _1^2}{\mathfrak {a}_2}\left( 2-\textit{m}^2\pm \sqrt{\textit{m}^4-\textit{m}^2+1}-3~ \text {dn}^2[\varrho ]+3~(-1+\textit{m}^2)~\text {nd}^2[\varrho ]\right) , \end{aligned}$$

where $$\varrho =\left( \rho _1 x+\rho _2 y+\rho _3 z+\rho _4 t\right)$$ and $$0\le \textit{m}\le 1$$.

**(6.3.5)** If $\textbf{b}_0=\frac{1}{4},\textbf{b}_2=\frac{1}{2}(\textit{m}^2-2)~\text {and}~\textbf{b}_4=\frac{\textit{m}^4}{4}$, the resulting solution exhibits a JE function form, as shown below:

62 $$\begin{aligned} \mathcal {T}_{6.3.5}=-\frac{ \rho _1^2}{\mathfrak {a}_2}\left( -4+2\textit{m}^2- \sqrt{\textit{m}^4-16\textit{m}^2+16}+3\,\frac{\textit{m}^4~\text {sn}^4[\varrho ]+(1+\text {dn}[\varrho ])^4}{ \text {sn}^2[\varrho ]\left( 1+\text {dn}[\varrho ]\right) ^2}\right) , \end{aligned}$$

where $$\varrho =\left( \rho _1 x+\rho _2 y+\rho _3 z+\rho _4 t\right)$$ and $$0\le \textit{m}\le 1$$.

When setting $\textit{m}=1$ in Eq.(62), the resulting solution exhibits a singular soliton form, as shown below:

63 $$\begin{aligned} \mathcal {T}_{6.3.5.1}=-\frac{ 3~\rho _1^2}{\mathfrak {a}_2} ~\left( 1+4\text {csch}^2 \left[ \;\left( \rho _1 x+\rho _2 y+\rho _3 z+\rho _4 t\right) \,\right] \right) . \end{aligned}$$

## Linear stability analysis

To investigate the stability of the steady-state solution of the (3+1)-dimensional P-type equation (1), we consider a small perturbation around a constant background. We write

$$\begin{aligned} \mathcal {T}(x,y,z,t) = \gamma \,\mathcal {P}(x,y,z,t) + \mathcal {R}, \end{aligned}$$

where $\gamma$ is the nonlinearity coefficient, $\mathcal {R}$ is the constant background, and $\mathcal {P}$ represents the perturbation. Substituting this ansatz into Eq. (1) and linearising with respect to $\mathcal {P}$ (neglecting terms of order $\mathcal {P}^2$ and higher) yields the linearised equation

64 $$\begin{aligned} \gamma a_4 \frac{\partial ^2 \mathcal {P}}{\partial z^2} + \gamma a_1 \frac{\partial ^2 \mathcal {P}}{\partial y \partial t} + \mathcal {R}\gamma a_2 \frac{\partial ^2 \mathcal {P}}{\partial x \partial y} + \gamma a_3 \frac{\partial ^2 \mathcal {P}}{\partial x^2} + \gamma \frac{\partial ^4 \mathcal {P}}{\partial x^3 \partial y} = 0. \end{aligned}$$

We look for plane-wave solutions of the form

$$\begin{aligned} \mathcal {P} = \exp \!\bigl (i(L_1 x + L_2 y + L_3 z) + \varpi t\bigr ), \end{aligned}$$

where $L_1, L_2, L_3$ are the wavenumbers in the *x*, *y* and *z* directions respectively, and $\varpi$ is the complex growth rate. Inserting this into Eq. (64), we obtain

65 $$\begin{aligned} \varpi = -\,\frac{i\Bigl (a_3 L_1^2 + L_1(\mathcal {R} a_2 - L_1^2)L_2 + a_4 L_3^2\Bigr )}{a_1 L_2}. \end{aligned}$$

**Fig. 1:**
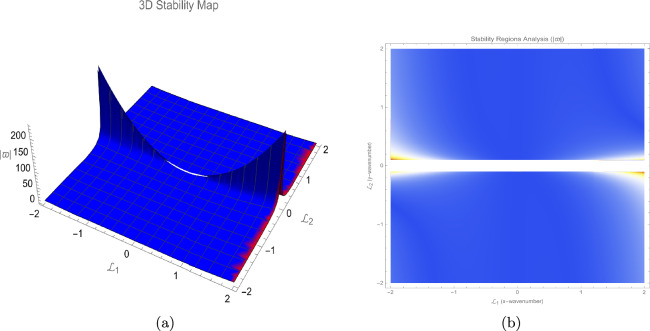
Linear stability analysis results.

Eq. (65) shows that $\varpi$ is purely imaginary for all real values of the parameters $a_1, a_2, a_3, a_4, \mathcal {R}$ and wavenumbers $L_1, L_2, L_3$ (provided $a_1 L_2 \ne 0$). Consequently, the perturbation $\mathcal {P}$ does not grow or decay exponentially in time; it only oscillates. The steady-state solution is therefore **neutrally stable** (marginally stable) within the framework of linear stability analysis. No instability occurs for any choice of the system parameters, and the constant background $\mathcal {R}$ does not trigger any exponential growth. It is worth noting that the special cases $L_2 = 0$ or $a_1 = 0$ require separate treatment. For $L_2 = 0$ the perturbation has no *y*-dependence; the linearised equation then reduces to a form that may admit different stability behaviour, which could be examined by a modified ansatz. Similarly, if $a_1 = 0$ the dispersion relation becomes algebraic in $\varpi$ and the dynamics become degenerate. In the generic case $a_1 L_2 \ne 0$, however, the system exhibits only oscillatory perturbations, indicating that the original equation (1) supports stable wave propagation without modulation instability. This can be illustrated in Fig. 1.

## Graphical representation

**Fig. 2:**
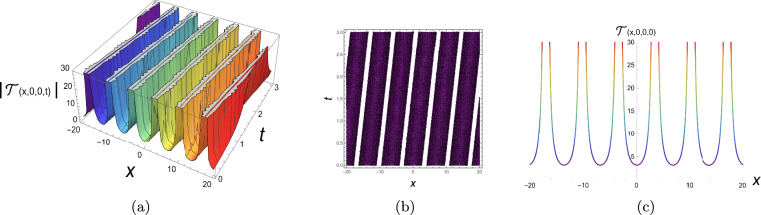
3D, contour and 2D diagrams of singular periodic solution corresponding to Eq. (10).

**Fig. 3:**
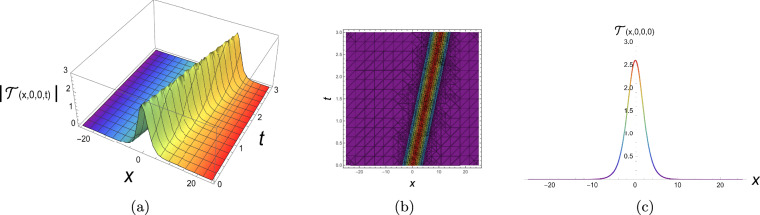
3D, contour and 2D diagrams of bright soliton solution corresponding to Eq. (12).

**Fig. 4:**
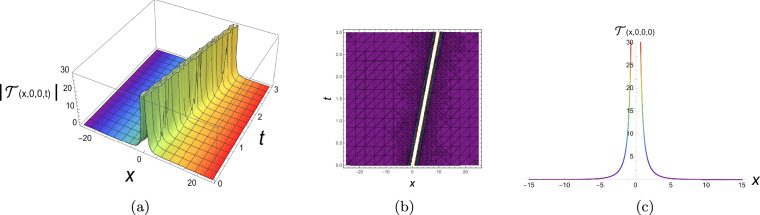
3D, contour and 2D diagrams of singular soliton solution corresponding to Eq. (18).

**Fig. 5:**
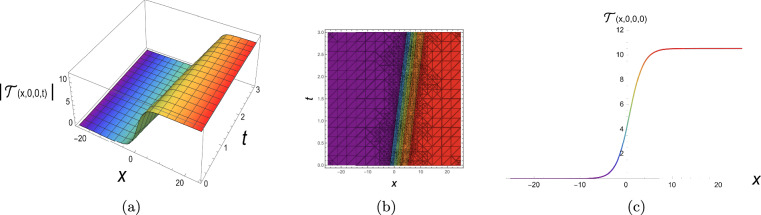
3D, contour and 2D diagrams of dark soliton solution corresponding to Eq. (29).

**Fig. 6:**
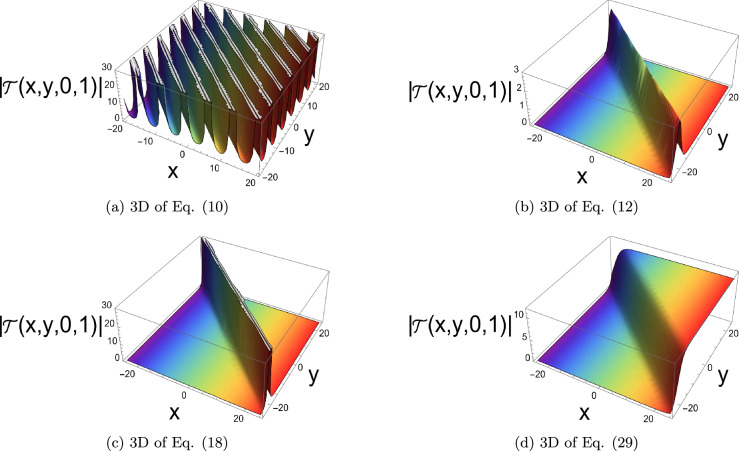
3D plots of the selected solutions in case $z=0$ and $t=1$.

**Fig. 7:**
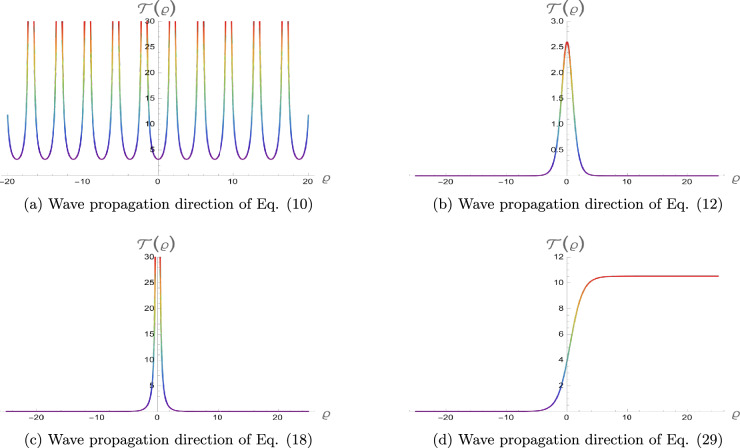
Wave propagation directions of the selected solutions versus $\varrho$..

The physical representation’s numerical simulations of some selected solutions in the form of 3D, contour, and 2D diagrams are displayed in this section. Figure 2 simulates a singular periodic solution corresponding to Eq. (10) when supposing ${\rho _1} = 0.55,\;{\rho _2} = 0.6,\;{\rho _3} = 0.65,\;{b_2} = - 0.7,\;$ ${\mathfrak{a}_1} = 0.75,\;{\mathfrak{a}_2} = - 0.8,\;{\mathfrak{a}_3} = 0.85,\;{\mathfrak{a}_4} = 0.9,\;y = 0,\;z = 0$ and x from −25 to 25. The solution exhibits a periodic sequence of sharply localized peaks, indicating strong spatial localization with no constant background level. The repeating structure reflects nonlinear wave propagation with pronounced confinement. Figure 3 simulates a bright soliton solution corresponding to Eq. (12) when supposing ${\rho _1} = 0.5,\;{\rho _2} = 0.55,\;{\rho _3} = 0.6,\;{b_2} = 0,65\;$ ${\mathfrak{a}_1} = 0.7,\;{\mathfrak{a}_2} = 0.75,\;{\mathfrak{a}_3} = 0.8,\;{\mathfrak{a}_4} = 0.85,\;y = 0,\;z = 0$ and x from −25 to 25. These plots demonstrate a single localized pulse on a zero background, maintaining its shape during propagation. This confirms stable balance between nonlinearity and dispersion. Figure 4 simulates a singular soliton solution corresponding to Eq. (18) when supposing ${\rho _1} = 0.6,\;{\rho _2} = 0.65,\;{\rho _3} = 0.7,\;{b_2} = - 0.75,$ ${\mathfrak{a}_1} = 0.8,\;{\mathfrak{a}_2} = 0.85,\;{\mathfrak{a}_3} = 0.9,\;{\mathfrak{a}_4} = 0.95,\;y = 0,\;z = 0$ and x from −25 to 25. The solution is highly localized with a sharp amplitude peak, indicating strong energy concentration and singular behavior, with no finite background level. Figure 5 simulates a dark soliton solution corresponding to Eq. (29) when supposing ${\rho _1} = 0.7,\;{\rho _2} = 0.75,\;{\rho _3} = 0.8,\;{b_2} = 0.85,$ ${\mathfrak{a}_1} = 0.9,\;{\mathfrak{a}_2} = 0.95,\;{\mathfrak{a}_3} = 1.05,\;{\mathfrak{a}_4} = 1.1,\;y = 0,\;z = 0$ and x from −25 to 25. These plots show a localized dip in amplitude on a nonzero background, characteristic of a stable dark soliton propagating without change in form. ToThese plots show a localized dip in amplitude on a demonstrate the genuinely three-dimensional nature of the plotted solutions, an additional plot in the (*x*, *y*)-plane (with fixed *z* and *t*) is presented in Figs. 6, 7 for each solution respectively, showing the variation of the wave profile in multiple spatial directions.

## Conclusion

We effectively discovered several types of exact wave solutions for the generalized (3+1)-dimensional P-type equation using the modified extended mapping method. As a result, the proposed model has several solutions, notably those for the (bright, dark, singular) soliton, JE function, exponential, and singular periodic solutions. In addition, a linear stability analysis was carried out, revealing that the steady-state solutions are neutrally stable for generic wavenumbers, with no exponential growth of perturbations. To help visualize how the equations behave dynamically, some figures are presented. The obtained solutions may be substantial and imperative for scrutinizing the phenomena arising in nonlinear science disciplines.

## Data Availability

The datasets used and/or analyzed during the current study are available from the corresponding author upon reasonable request.
